# Advancing the understanding of palliative care in Singapore: knowledge, attitudes, receptiveness, and the moderating role of media information-seeking preferences

**DOI:** 10.3389/fpubh.2026.1774749

**Published:** 2026-04-02

**Authors:** Su Lin Yeo, Angel Lee, Raymond Han Lip Ng, May O. Lwin, Yumin Lin

**Affiliations:** 1 Lee Kong Chian School of Business, Singapore Management University, Singapore, Singapore; 2 St. Andrew’s Community Hospital, Singapore, Singapore; 3 Palliative and Support Care, Woodlands Hospital, Singapore, Singapore; 4 Wee Kim Wee School of Communication & Information, Nanyang Technological University, Singapore, Singapore; 5 Asian Centre for Health Behavioural Insights & Interventions (HABITS), Nanyang Technological University, Singapore, Singapore

**Keywords:** attitude, end-of-life, information seeking, knowledge, palliative care, receptiveness, traditional and digital media

## Abstract

**Introduction:**

Despite the growing demand for palliative care, this specialized medical care remains severely underutilized, with almost 50% of countries worldwide lacking access. Given that misperceptions and stigma continue to hinder progress in healthcare policy, this study aims to examine the relationships between palliative care knowledge, attitudes, and receptiveness to better understand how public health communication can enhance awareness and understanding of the topic in Singapore. Applying the adapted knowledge-attitude-practice (KAP) model, it further extends theory by examining the moderating role of media information-seeking between knowledge and receptiveness.

**Methods:**

Mixed-mode surveys involving 1,226 participants (926 online and 300 in-person respondents), representing various population groups, were carried out in the city-state. To ensure a representative sample of the general population, data from diverse age groups from 21 to 61 years and above were collected to provide a broad and accurate assessment based on five hypotheses guided by our theoretical model. Our respondents’ knowledge of palliative care, attitudes toward end-of-life care planning, receptiveness to palliative care, information-seeking preferences, and demographic profiles were assessed and statistically analyzed using the PROCESS macro in SPSS.

**Results:**

Our findings validated the KAP model (adapted), which suggested that a higher level of knowledge is associated with a more positive attitude, which in turn has a positive impact on receptiveness. Our study further found that the relationship between palliative knowledge and receptiveness was stronger among individuals who frequently acquired information via digital media; however, this relationship did not hold for those who acquired information via traditional media.

**Conclusion:**

Our findings concurred with previous studies that validated the KAP model used to investigate other public health issues among healthcare professionals. Given that the literature is limited on the validation of this model on the acquisition of palliative care knowledge among population groups, this study closes critical knowledge gaps by being one of the few studies to offer insights into developing effective media strategies needed for public health communication and education to increase awareness of palliative care within a country’s population.

## Background

As many populations around the world age rapidly, the global need for palliative care will only continue to increase. By 2050, the number of people aged 60 years and over is expected to double from 1 billion in 2019 to 2.1 billion (1). The rising burden of terminal and life-threatening illnesses demands the need for health authorities to offer person-centered and integrated health services in order to support ailing individuals and their families as they face challenging end-of-life (EOL) issues. A medical approach that addresses issues such as physical suffering, psychological, social, and spiritual distress, and early delivery of palliative care has been shown to improve the quality of life of patients and their caregivers throughout the entire duration of patients’ advanced illnesses until the end of life (2).

Despite medical evidence demonstrating the advantages of palliative care, understanding, acceptance, and utilization of this service remain low among many populations. Earlier studies have shown that nearly 50% of countries worldwide lack access to the service (3, 4). While 56.8 million people worldwide need to receive palliative care every year, only 14% of them utilize it (1). Its slow delivery and development have been attributed to a poor understanding among healthcare professionals and terminally ill patients in developing countries (5). In developed countries, inaccurate perceptions of quality EOL care persist among the general population (6). For decades, scholars have attributed the lack of knowledge and the reluctance to talk about death as barriers to promoting public awareness of palliative care (7). Additional factors that hinder the provision of care include insufficient education for patients and care providers, cultural differences, attitudes toward EOL planning, and preferences for more life-sustaining treatment (8–10).

Despite international efforts to foster greater public awareness within healthcare communities over the past decades (11), findings from recent studies continue to reveal knowledge gaps, persistent negative perceptions, and low utilization of palliative care services among populations worldwide (12–14). As family members often make decisions for patients toward the end of life, those who misconceive the benefits of palliative care may not discuss relevant EOL issues with their terminally ill loved ones. As a result, the care needed is either delayed or denied (15). Poor knowledge remains one of the most common barriers to palliative care acceptance (16), and common misperceptions of this medical approach include “giving up” and death (17). Such stigmatization of palliative care has led to decreased intentions to engage in palliative care for oneself and one’s family members (18).

Considering that death is an unavoidable life event that requires conversations on medical treatments and transition to palliative care, it is imperative for individuals facing life-threatening illnesses to engage in discussions about EOL issues with their loved ones alongside their information and decision-making preferences (19). This underscores the need to adequately inform the general population about advance care planning, treatment options, and personal values connected to death and dying (20). However, the rapid growth and aging of the population have created palliative care needs that cannot be addressed by healthcare professionals alone (21, 22). Ensuring equitable access to quality palliative care, therefore, requires extending discussions beyond clinical settings into the broader domain of public health communication. It is only when health authorities make EOL issues a national priority and strategically disseminate public health messages that this will encourage conversations on death and proactive information-seeking behaviors leading to increased knowledge, attitude shifts, and improved receptiveness to palliative care (23, 24).

### A super-aged Singapore society in 2026

Singapore, a highly globalized Southeast Asian city-state, has a rapidly aging population (25). With declining fertility and rising life expectancy, the society is now super-aged, and one in four residents will be aged 65 or older by 2030 (26). As cancer and chronic non-communicable diseases remain the leading causes of death in Singapore, older adults will be diagnosed with these diseases in the future. They will require long-term care until death (27). However, Singaporean palliative care service providers are struggling to keep pace with the rate of societal aging. For instance, many healthcare workers in Singapore feel inadequate to provide end-of-life care (28). Knowledge gaps and evolving perceptions of palliative and hospice care may explain why few Singapore physicians would consider referral at the initial diagnosis of a life-limiting illness (29). As misperceptions exist at all levels of medical training and experience, palliative care scholars are advocating for effective national education and stakeholder engagement to advance understanding and increase palliative care knowledge among healthcare professionals in Singapore (30).

As for Singapore’s public perceptions and awareness of palliative care, findings were equally dismal. A recent national survey found that the general population lacks understanding of EOL care, as only 53% of Singapore residents have heard of hospice palliative care (31). Across all segments of society, research to date suggests that a lack of knowledge about palliative care is the greatest barrier to understanding the role and utilization of EOL care services. With the rapidly growing population of older adults in the city-state, if public health communicators do not quickly address this inadequacy, the widening gap between knowledge and receptiveness of palliative care will only escalate medical costs, burden healthcare systems, and deny individuals’ right to quality palliative care.

Therefore, this study answers Tullis’ call for communication to play a greater role in interdisciplinary research to better inform health practices (32). We are convinced that this research is timely because decades of palliative care research have yet to offer practical solutions to raise public awareness and increase knowledge, thereby altering attitudes toward greater acceptance among population groups. Echoing the sentiments of Ragan et al. (33), it is essential for the medical field to integrate palliative care literature with health communication scholarship in order to break down the “hardened” barriers that currently limit access to these vital services so that no individual needs to die and suffer in pain at the end of life.

Drawing on theoretical insights from the knowledge-attitude-practice (KAP) model in motivational behavior, this study proposes to investigate the relationships between palliative care knowledge, attitudes toward EOL care planning, and receptiveness of palliative care among the Singapore population. While this model has been used to examine various health-related behavioral changes by healthcare providers, the literature is scant regarding its application and validation in the acquisition of palliative care knowledge within a country’s population, which would lead to attitude shifts and receptiveness to palliative care in three successive processes. This study further extends the KAP model by investigating a moderating variable—media information-seeking preferences—to determine if this factor changes the relationship between knowledge and receptiveness. Given the dearth of palliative care communication in health communication literature, we aim to close significant knowledge–practice gaps and provide insights to public health authorities worldwide who are seeking solutions to increase their knowledge of population’s palliative care in the digital economy.

### Knowledge-attitude-practice (KAP) model: attitude as a mediator between knowledge and receptiveness

The KAP framework, a behavioral change model, offers a methodical approach to understanding health behaviors. It is frequently used in public health research by healthcare scholars seeking to design effective health messages to resonate with a target audience’s level of understanding (34). Originating from both the learning theory (35) and the diffusion of innovations (36), the model addresses “the central theoretical problem in the field of purposive communication is explaining the gap between knowledge and behavior” (37). The model suggests a linear relationship between knowledge, attitudes, and practice, indicating that the three constructs are related. This is because individuals who are highly knowledgeable about an issue are expected to develop a positive attitude toward it. This positive attitude will thereafter manifest in their behavior and actions (38). In sum, it posits a logical sequence of behavioral change, maintaining that people first learn about a certain behavior, which subsequently leads them to develop a favorable attitude toward it, thereby motivating them to practice it (39).

Over the decades, the KAP model has been adapted to incorporate similar or subdimensions of the construct, “practice,” depending on the end outcome of the measurement, which may not necessarily be about tangible practice or visibly manifested behaviors. Some healthcare researchers have replaced “practice” with “beliefs,” (40) “preferences,” (41) “perceptions,” (42) or “receptiveness” (43) in the behavioral process, although the ordering of knowledge–attitude–practice remained largely the same (44). For our study, which aims to better understand individuals’ willingness to accept taboo medical services such as palliative care, we will use the term “receptiveness” in place of “practice” as receptiveness is more reflective of the resolute end outcome of accepting EOL care (16). Being receptive to palliative care is also not akin to “practice” as it is hard to measure actual behavior since it can only occur with doctors’ recommendations (45).

Although only some earlier studies have examined the association between palliative care knowledge and receptiveness, researchers found that those who were knowledgeable reported more positive perceptions (46). For example, Baker reported that healthcare workers with greater knowledge of advance care planning tended to hold a favorable attitude toward providing the service (47). Therefore, we aim to examine the relationships between these three variables, namely (1) knowledge of palliative care, (2) attitudes toward EOL care planning, and (3) receptiveness to receiving palliative care. To validate the ordering of the dimensions, we hypothesize that the following:

*H1*: Palliative care knowledge will be positively related to palliative care receptiveness.

*H2*: Attitudes toward EOL care planning will be positively related to palliative care receptiveness.

*H3*: Attitudes toward EOL care planning will mediate the relationship between palliative care knowledge and receptiveness.

### Media information-seeking preferences as a moderator in knowledge-attitude-receptiveness

In addition to testing the relationship between knowledge, attitudes, and receptiveness, we propose to include media information-seeking preferences as a moderator. Information-seeking in health communication literature refers to the intentional pursuit of information through selected channels to guide decision-making (48) and often functions as a coping strategy to reduce uncertainty (49) among patients with cancer (50). In palliative care, life-threatening diagnoses create profound uncertainty for patients and their families (51), heightening psychological distress and the risk of maladaptive decisions (52). To address these gaps, they seek information from multiple sources, including physicians (53, 54) and media channels (55).

Health communication literature has consistently demonstrated that media play a pivotal role in delivering health information (56). Traditionally, the public uses mass media as the primary source to raise awareness and make health decisions (57). Individuals who seek information from traditional media have been found to be more knowledgeable about the need to engage in healthy activities (58, 59). In recent times, with the advent of the Internet, a virtual public sphere has emerged, providing extensive access to health information through online connections and digital platforms (60). As uncertainty is often prevalent in health decision-making and can magnify anxiety and negative coping (52), the interactive nature of digital media may provide more social support and emotional relief, leading to positive behavioral outcomes (61). Studies have demonstrated that, among patients with chronic and EOL conditions, seeking information from digital media has helped to generate comfort (62), increase knowledge (63), and enhance protective behavioral intentions (64).

However, the literature is scarce on how media-based information-seeking intentions influence knowledge and receptiveness to palliative care within a population. As we aim to close knowledge gaps by including media information-seeking preferences, i.e., traditional and digital media, as a moderator to better comprehend the effects of palliative care knowledge on receptiveness, we further posit that the following hypotheses:

*H4*: Palliative care information seeking via traditional media will moderate the relationships between palliative care knowledge and receptiveness so that the association between knowledge and receptiveness is stronger for individuals who seek and consume more information from traditional media.

*H5*: Palliative care information seeking via digital media will moderate the relationships between palliative care knowledge and receptiveness so that the association between knowledge and receptiveness is stronger for individuals who seek and consume more information from digital media.

## Methods

### Sample and design

A cross-sectional survey was conducted to ensure that our sample was nationally representative of the general population. First, 926 online respondents were recruited by an academic research company between April and July 2019 to complete a 20-min online survey. The participants were Singapore citizens or permanent residents aged 21 to 60 years who were proficient in English and digitally savvy. Second, another 300 in-person participants (non-English speakers who are not digitally savvy) were recruited between July and August 2019. They were recruited offline by another academic research organization that purchased a sampling database from official statistics representing different addresses and districts in Singapore. The inclusion criteria for this group were as follows: (1) aged 51 years or older, (2) limited English proficiency, and (3) limited digital proficiency. The questionnaire from the online survey was translated from English into Chinese by three on-field translators through multiple rounds of backtranslation and cross-checking. For data collection, we trained seven effectively bilingual interviewers in (a) English and Mandarin, (b) English and Malay, and (c) English and Tamil; and they were subsequently matched with the participants’ language preferences. During the door-to-door survey, the interviewers were required to (a) convey questions and options clearly; (b) confirm that the participants had understood every question before recording their responses on the survey form; and (c) thank the participants for their time before presenting them with an incentive at the end of the survey. After the data were collected, 10% of the participants from each interviewer were contacted by the researchers to verify that the surveys were conducted in accordance with our training guidelines. Each in-person participant took approximately 35 min to complete the survey.

Mixed-mode surveys have gradually become an effective and acceptable approach in research, particularly for data collection across respondents with varied characteristics (65). The combination of online and door-to-door recruitment of respondents enabled the collection of data from a representative sample of the Singaporean population. Data collected solely from online surveys faced challenges related to external validity, as the skills and access required for participation in online surveys could be an issue for certain population groups (66). For example, a 2023 study by the SMU Centre for Research on Successful Ageing reported structural and attitudinal obstacles faced by seniors in Singapore who are technologically challenged (67). Hence, the mixed-mode survey enables the inclusion of older adults who do not use digital technologies in the study sample, thereby increasing the external validity of our research. As expected, the sample recruited online in our study demonstrated significantly better palliative care knowledge [*t*(1224) = −9.63, *p* < 0.001], more favorable attitudes toward EOL care planning [*t*(1224) = −4.94, *p* < 0.001], palliative care receptiveness [*t*(1224) = −3.47, *p* = 0.001], and information-seeking from digital media [*t*(1224) = −29.45, *p* < 0.001], as compared to the sample recruited door-to-door (see Supplementary material). These significant differences supported the necessity to employ the mixed-mode survey.

In total, our sample comprised 1,226 respondents. As a multi-ethnic society, we further ensured that the ethnic composition of the Singaporean population reflected national representation (74.3% Chinese, 13.4% Malays, 9.0% Indians, and 3.2% others) (68). Approval from the Institutional Review Board (IRB) at the Singapore Management University was obtained before data collection. We also wish to declare that the data analyzed in this study were part of a larger set of data collected nationally. Portions of the same dataset have previously been used to report the public sentiments on the same topic in Singapore. However, the earlier study investigated the direct effects of interpersonal and media information-seeking (and their sub-dimensions) on multiple outcomes (31), whereas the present study addresses a distinct theoretical question by testing a moderated mediation model to explain the various relationships between palliative care knowledge and receptiveness within the KAP framework.

### Measures

#### Palliative care knowledge

We created a six-item measure for palliative care knowledge based on input from local healthcare professionals in palliative and hospice sectors. Participants were asked to indicate whether each statement was true on a 3-point scale (1 = *No*, 2 = *Not sure*, 3 = *Yes; “Yes”* to be the correct answer for all). The sum of the scores was used as an indicator of participants’ palliative care knowledge, with higher scores reflecting better knowledge (Cronbach’s *α* = 0.77, *M* = 14.98, SD = 2.56).

#### Information-seeking from traditional media

Participants were asked to indicate their likelihood of using (1) newspapers, (2) magazines, (3) radio, and (4) television to obtain information about palliative care, on a five-point scale ranging from 1 (*Extremely unlikely*) to 5 (*Extremely likely*) (69). The scores from the four items were averaged to indicate participants’ information-seeking from traditional media (Cronbach’s *α* = 0.88, *M* = 2.98, SD = 1.00).

#### Information-seeking from digital media

Similar to the measures for traditional media, the participants were asked to indicate their likelihood of using (1) search engines on the Internet and (2) social media (e.g., Facebook and X) to obtain information about palliative care, on a five-point scale (69). The mean scores of the two items were used to indicate participants’ information-seeking from traditional media (Cronbach’s *α* = 0.74, *M* = 3.32, SD = 1.13).

#### Attitudes toward EOL care planning

We adapted the seven-item measure to assess participants’ attitudes toward EOL care planning (70). The answers were marked on a five-point scale (1 = *Strongly disagree*, 5 = *Strongly agree*). Composite scores were computed using the average score, and a higher score reflected a more favorable attitude (Cronbach’s *α* = 0.82, *M* = 3.76, SD = 0.55).

#### Palliative care receptiveness

We created a three-item measure to capture participants’ likelihood of considering palliative care for different target populations (i.e., “for yourself,” “for family members,” and “for close friends”) on five-point scales (1 = *Highly unlikely*, 5 = *Highly likely*). The mean of these three items indicated receptiveness, and a higher score suggested a higher likelihood of considering palliative care (Cronbach’s *α* = 0.83, *M* = 3.36, SD = 0.79).

#### Demographics

Participants’ sociodemographic profiles were also assessed, including age, gender, ethnicity, marriage, education, monthly household income, housing type, language, and current health status.

### Statistical analysis

**Figure 1:**
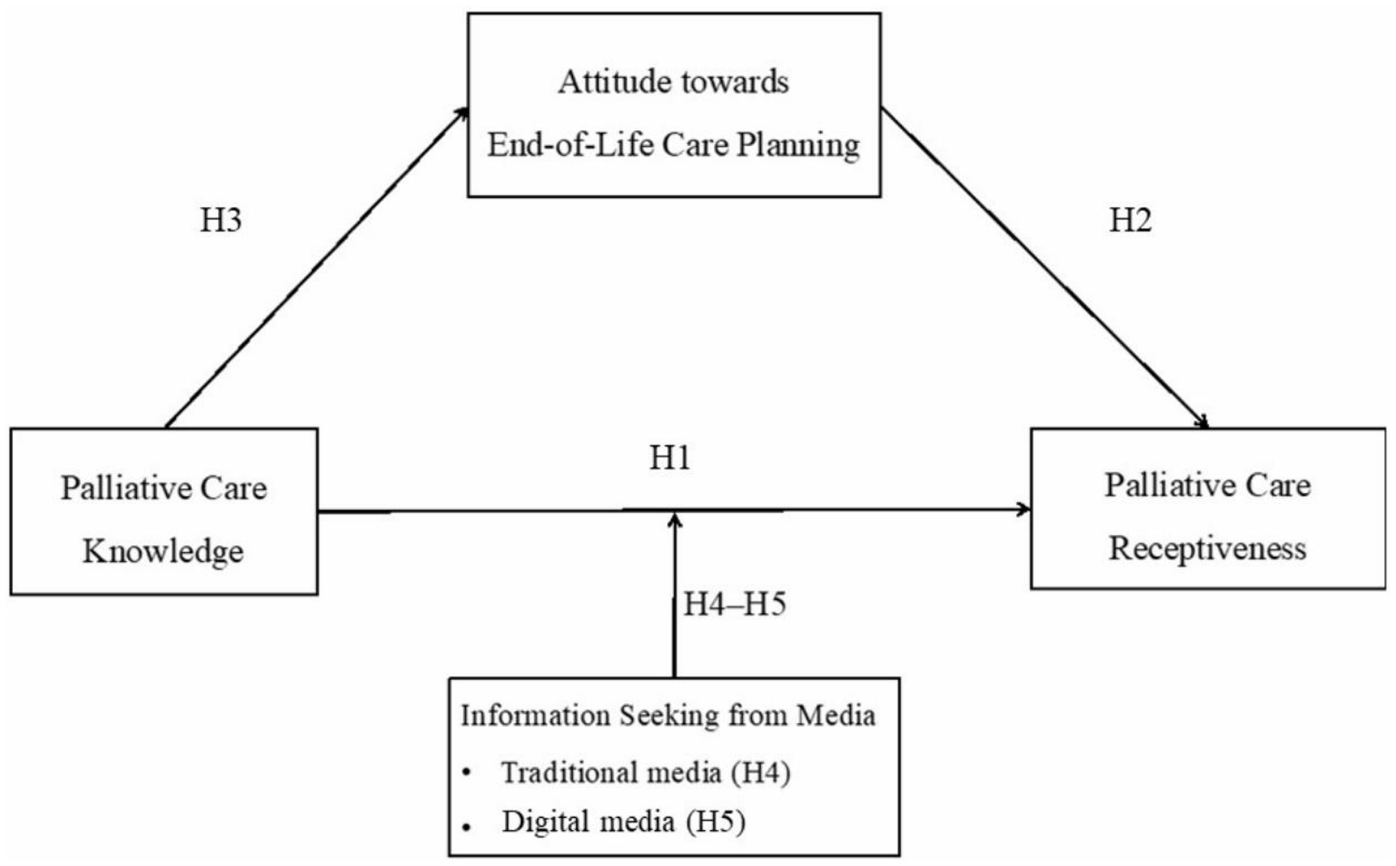
Hypothesized model. H1–H5, Hypothesis 1–5.

We analyzed the data using SPSS (Version 25; IBM, New York, NY). Descriptive statistics were used to analyze demographics, including age, gender, ethnicity, marital status, education, household income, housing type, language, and health status. Independent-samples *t-*test, one-way ANOVA, and Pearson correlation were performed to examine whether demographic factors were associated with disparities in receptiveness to palliative care. A mediation analysis was thereafter conducted using the PROCESS macro in SPSS (Model 4) to examine the hypothesized relationships between knowledge, attitudes, and receptiveness (H1–H3; see Figure 1) (71). Furthermore, to examine the extent to which media information-seeking preferences moderated knowledge and receptiveness (H4–H5; also see Figure 1), two sets of moderation analyses were conducted using the PROCESS Model 5. Through these analyses, the relationships between all variables were estimated. The bootstrapping method was applied using 5,000 samples to test the significance and generate a valid 95% confidence interval (71, 72). The use of bootstrapping was necessary to minimize the impact of the shape of the sampling distribution of the data when running inferential analyses (72). Demographic factors were controlled in the mediation and moderation analyses.

## Results

### Sample characteristics

Among 1,226 participants, 49.8% were men, and 50.2% were women. The ages ranged from 21 to 61 years and above. Table 1 summarizes the basic demographic information of the sample, with the majority of the participants being ethnically Chinese (76.6%) and having a university degree or higher (47.5%).

**Table 1:** Demographic profile of the recruited sample in the study.

Variable	Group	*n*	%
Gender	Male	610	49.8
	Female	616	50.2
Age	21–30	274	22.3
	31–40	311	25.4
	41–50	202	16.5
	51–60	249	20.3
	≥ 61	190	15.5
Ethnicity	Chinese	939	76.6
	Malay	159	13.0
	Indian	94	7.7
	Others	34	2.8
Education	No formal schooling	109	8.9
	PSLE or equivalent	119	9.7
	GCE O/N level	166	13.5
	GCE A-level/Diploma	250	20.4
	Degree/Higher education	582	47.5

*N* = 1226.

PSLE, Primary School Leaving Examination; GCE O-Level, Singapore-Cambridge General Certificate of Education Ordinary Level; GCE N-Level, Singapore-Cambridge General Certificate of Education Normal Level; GCE A-level, Singapore-Cambridge General Certificate of Education Advanced Level.

### Mediating role of attitude between knowledge and receptiveness (H1–H3)

Hypothesis 1 predicted that palliative care knowledge would affect receptiveness. As shown in Table 2, participants’ knowledge of palliative care was positively related to their receptiveness (*β* = 0.12, *p* < 0.001). Thus, the more people knew about palliative care, the more likely they would consider palliative care in the future, supporting H1.

**Table 2:** Mediation effect of attitudes on the link between knowledge and receptiveness (H1–H3).

Predictor	*β*	SE	*t*	*p*
(DV = Attitude)				
Knowledge	0.16***	0.01	5.58	0.000
(DV = Receptiveness)				
Knowledge	0.12***	0.01	4.04	0.000
Attitude	0.19***	0.04	6.52	0.000

Predictor	*B*	Bootstrap SE	Bootstrap 95% CI
(DV = Receptiveness)			
Indirect effect of Knowledge	0.01*	0.002	[0.005, 0.015]

*N* = 1226. *p < 0.05, **p < 0.01, ***p < 0.001. Bootstrap sample size = 5,000.

CI, confidence interval; DV, dependent variable; SE, standard error. Demographic variables were controlled.

Hypothesis 2 predicted that attitudes toward EOL care planning were positively related to receptiveness. As expected, the results showed a positive relationship between attitudes and receptiveness (*β* = 0.19, *p* < 0.001). Hence, H2 is supported.

Hypothesis 3 posited that the link between palliative care knowledge and receptiveness would be mediated by attitudes toward EOL care planning. To examine this mediation effect more closely, the bootstrapping approach was used to generate a 95% confidence interval. The results showed that the indirect effect of knowledge on receptiveness (via attitude) was positively significant (*B* = 0.01, SE = 0.002, 95% CI = 0.005, 0.015). It indicated that higher levels of knowledge are associated with higher levels of attitude and higher levels of receptiveness. Thus, H3 is also supported.

### Moderating role of information-seeking from traditional and digital media (H4–H5)

**Figure 2:**
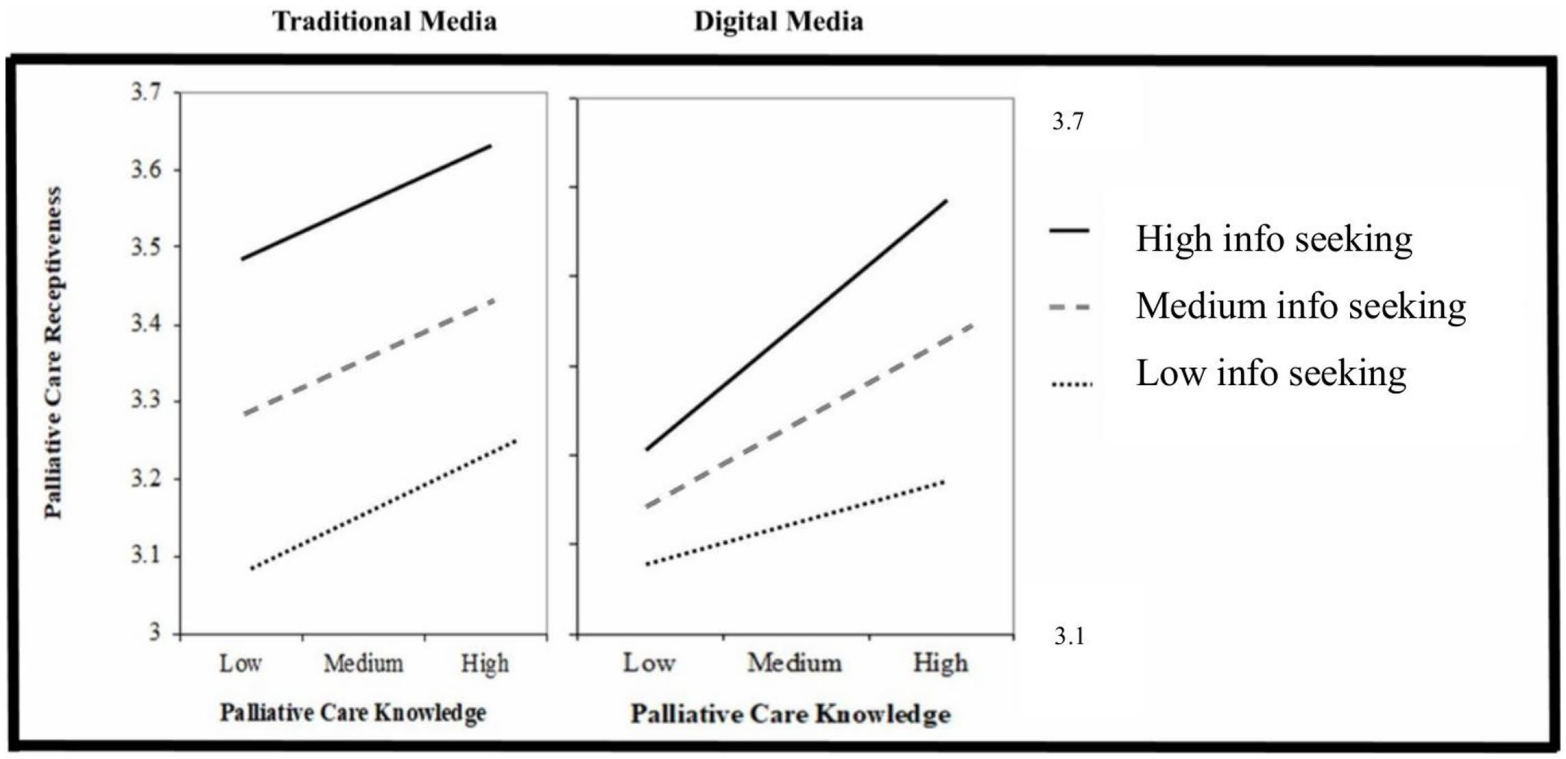
Moderation roles of information seeking from different sources. Information seeking from traditional media not moderating the effect of palliative care knowledge on receptiveness (*p* 0 > 0.05). Information seeking from digital media moderating the effect of palliative care knowledge on receptiveness (*p* < 0.05).

Hypothesis 4 predicted that individuals’ information-seeking from traditional media would serve as a positive moderator between palliative care knowledge and receptiveness. As shown in Table 3, the interaction term Knowledge × Info-seeking from traditional media was not significant (*p* = 0.83 > 0.05). To better illustrate the non-significant results, the relationship between knowledge and receptiveness was plotted at low, medium, and high levels of traditional media preferences and showed no noticeable variation in the pattern (Figure 2). Thus, H4 is not supported.

**Table 3:** Moderation effect of information seeking from traditional media (H4).

Predictor	*β*	SE	*t*	*p*
(DV = Receptiveness)				
Attitude	0.18***	0.040	6.36	0.000
Knowledge	0.09**	0.01	3.18	0.001
Information Seeking from Traditional Media	0.26***	0.02	9.31	0.000
Knowledge **×** Information Seeking from Traditional Media	n.s.	0.01	−0.22	0.83

*N* = 1226. *p < 0.05, **p < 0.01, ***p < 0.001. n.s., not significant. Bootstrap sample size = 5,000.

CI, confidence interval; DV, dependent variable; SE, standard error. All predictors were mean-centered before analysis. Demographic variables were controlled.

Hypothesis 5 predicted that information-seeking from digital media would moderate the relationship between knowledge and receptiveness. The results showed that the interaction term Knowledge × Info-seeking from digital media was statistically significant (*β* = 0.07, *p* < 0.05, see Table 4), confirming a moderation effect. Therefore, H5 is supported.

**Table 4:** Moderation effect of information seeking from digital media (H5).

Predictor	*β*	SE	*t*	*p*
(DV = Receptiveness)				
Attitude	0.17***	0.04	5.64	0.000
Knowledge	0.12***	0.01	3.87	0.000
Information seeking from digital media	0.14***	0.03	3.90	0.000
Knowledge **×** information seeking from digital media	0.07*	0.01	2.36	0.019
Conditional effects of knowledge at values of information seeking from digital media (DV = Receptiveness)				
Low information seeking from digital media (mean − 1 SD)	n.s.	0.01	1.35	0.179
Medium information seeking from digital media (mean)	0.12***	0.01	3.87	0.000
High information seeking from digital media (mean + 1 SD)	0.20***	0.01	4.24	0.000

*N* = 1226. *p < 0.05, **p < 0.01, ***p < 0.001. n.s., not significant. Bootstrap sample size = 5,000.

CI, confidence interval; DV, dependent variable; SE, standard error. All predictors were mean-centered before analysis. Demographic variables were controlled.

Conditional effects of knowledge on receptiveness were further examined at three values of information-seeking from digital media (low, medium, and high). Our findings showed that the relationship between knowledge and receptiveness became stronger when people had a greater preference to seek palliative care information from digital media. As for individuals who rarely relied on digital media, their knowledge was no longer a significant predictor of receptiveness (*p* = 0.179 > 0.05, see Table 4). To better illustrate these relationships, a moderation plot was generated (Figure 2), suggesting that digital media served as a positive moderator, strengthening the relationship between palliative care knowledge and receptiveness, whereas traditional media did not.

## Discussion

Our study aims to achieve three objectives: establish the relationships between knowledge, attitudes, and receptiveness toward palliative care; validate the order of the three constructs as informed by the KAP model; and examine the moderating role of media information-seeking preferences in the relationship between knowledge and receptiveness. Our findings confirmed the relationship between palliative knowledge, attitudes, and receptiveness: knowledge was positively associated with receptiveness, both directly and indirectly through more favorable attitudes. This aligns with previous studies that validated the order of the three constructs when applied to other health behaviors. Furthermore, the positive relationship between palliative knowledge and receptiveness was stronger among individuals with higher information-seeking preferences from digital media. This moderated relationship, however, was not found for information-seeking from traditional media.

Our research showed that if Singapore aims to effectively prepare for the challenges posed by an aging population, public health communication should be strengthened to improve knowledge of palliative care. Coordinated communication programs should be devised to reach the public and targeted communities (including education in schools). Additionally, inter-group and inter-personal communication should be supported via various media platforms, with greater emphasis on digital and social media. In healthcare, high-quality decisions, such as knowledge-based ones, are made through deliberative reasoning; these high-quality medical decisions are often labelled as informed decisions (73). Sufficient knowledge and a positive attitude toward the decision often play essential roles in the construct of informed decision-making (74). In other words, improved knowledge and favorable attitudes enhance informed decision-making.

In the context of palliative care, our research provided empirical support for the mediating role of attitudes in the relationship between knowledge and receptiveness. The findings closed knowledge gaps by being one of the few studies to apply KAP in the context of EOL care on a population. It further supplemented empirical evidence from other studies guided by the Health Belief Model (75) by demonstrating the mediating role of the direct and parallel functions of knowledge and attitudes on behavioral intentions. External factors, such as pre-existing knowledge, could guide how individuals evaluate the health risks associated with a desired health behavior and influence their perception of the benefits and barriers before engaging in the health behavior.

As for the moderating role of media information-seeking preferences, it is not surprising that individuals prefer to seek information on palliative care from digital media. In the digital era, the Internet is often the first platform many turn to because it offers a wealth of information. For those facing life-threatening illnesses, including concerns about dying and death, seeking comfort from social online communities (even strangers) may provide a sense of closure. Taking to digital spaces can also help us address uncomfortable emotions that we would otherwise avoid in face-to-face conversations (76). Although information-seeking through traditional media did not play a significant moderating role, mainstream media coverage is nonetheless necessary to spark public conversations. One factor that may explain why seeking information from traditional media on palliative care was less significant than for infectious diseases like COVID-19 could be that traditional media often lack two-way engagement and practical guidance for individuals seeking tangible and emotional connections to make complex medical and personal decisions (77). To increase knowledge of palliative care, public health communication should first prioritize digital and social media, as individuals have been shown to prefer digital education on taboo subjects such as death (78–81). Knowledge-generating content, for instance, can take the form of creatively produced online videos, personal testimonies, and animated drawings that subtly explain EOL issues, positively shift individuals’ attitudes, and increase receptiveness to palliative care.

Although this study offers valuable insights, it has several limitations. First, in addition to knowledge and attitudes as predictors of receptiveness to palliative care, other highly predictive social factors may also influence receptiveness. Future studies should consider cultural and religious variables in advancing knowledge on palliative care or even measure actual behaviors. Second, despite being theoretically driven, this study cannot establish causal relationships between the examined variables. Further research should collect longitudinal data to better understand the temporal sequence of the key factors in the model. Additionally, with the development of generative AI tools, AI chatbots have gradually become an important source of health information (82). Future studies should also explore the role of information-seeking via AI chatbots in digital information-seeking behaviors.

Nevertheless, this study makes a theoretical contribution by validating the KAP model as an explanation for the acquisition of palliative care knowledge within a population. It provides empirical evidence to inform the development and implementation of media-based public health communication strategies aimed at promoting greater receptiveness to EOL and palliative care. These insights can further facilitate palliative care communication among vulnerable groups. By utilizing the KAP model to better understand and align information-seeking preferences to knowledge creation, vulnerable groups can benefit from different tailored media-driven communication approaches.

## Conclusion

Palliative care is an increasingly vital medical service as the global population ages and countries worldwide, including Singapore, undergo demographic shifts. Low levels of knowledge and unfavorable attitudes among populations caused by misperceptions, if allowed to remain, will only hinder acceptance of and receptiveness to EOL care. Theoretically driven research informed by established frameworks, such as the KAP model, provides a structured roadmap and empirical basis for effective public health education and communication, particularly in an increasingly dynamic digital and AI environment. Optimizing digital media to strengthen national education programs and social discussions can help improve public understanding of palliative care and begin to address the unique societal challenges caused by shifting disease burdens in Singapore and many other aging societies.

## Data Availability

The datasets presented in this article are not readily available because the datasets used and/or analyzed during the current study are available from the corresponding authors upon reasonable request. Requests to access the datasets should be directed to Su Lin Yeo, sulinyeo@smu.edu.sg.
