# Supplementary material for: Advancing the understanding of palliative care in Singapore: knowledge, attitudes, receptiveness, and the moderating role of media information-seeking preferences
Source: Front Public Health. 2026 Apr 2;14:1774749. doi: 10.3389/fpubh.2026.1774749 (PMC13083184; doi:10.3389/fpubh.2026.1774749)
Supplement: Supplementary file 1 [file Data_sheet_1.docx]

**Supplement**

*Table 5 Key Comparisons between Online and Door-to-door Samples*

|  |  | **Online**  ***n*=926** | **Door-to-door**  ***n*=300** |
| --- | --- | --- | --- |
| Palliative Care Knowledge | *M* | 24.36 | 22.21 |
|  | *SD* | 3.33 | 3.48 |
| Attitude towards End-of-life Care Planning | *M* | 3.80 | 3.62 |
|  | *SD* | .56 | .49 |
| Palliative Care Receptiveness | *M* | 3.40 | 3.22 |
|  | *SD* | .78 | .79 |
| Information Seeking from Digital Media | *M* | 3.74 | 2.05 |
|  | *SD* | .74 | 1.15 |

**Supplement**

***Measures of Key Variables in Study (English and Chinese)***

**# Palliative Care Knowledge**

What do you think Palliative Care is about?

您觉得慈怀疗护是什么？

|  | No  不是的 | Not sure  不确定 | Yes  是的 |
| --- | --- | --- | --- |
| (1) Care for those who are dying in the last stage of life.  照顾处于生命最后时光的人。 | ❍ | ❍ | ❍ |
| (2) Physical and psychological wellness of a terminally ill patient.  关心晚期病人的身体和心理健康。 | ❍ | ❍ | ❍ |
| (3) Helping the family to take care of patients who are living through the last stage of life.  帮助家庭去照顾正经历生命最后时光的病人。 | ❍ | ❍ | ❍ |
| (4) Care for elderly and children with life-limiting conditions. 照顾生命有限的老人和小孩。 | ❍ | ❍ | ❍ |
| (5) Management of distressing symptoms like pain and breathlessness.  控制危险的病情，比如：疼痛、呼吸困难。 | ❍ | ❍ | ❍ |
| (6) Providing psychological and emotional support for the patient. 慈怀疗护会向 病人 提供心理和情感支持。 | ❍ | ❍ | ❍ |
| (7) Providing psychological and emotional support for the family. 慈怀疗护会向 病人的家人 提供心理和情感支持。 | ❍ | ❍ | ❍ |
| (8) Includes helping with spiritual issues (e.g., Why do I have to suffer?) 慈怀疗护会帮助有精神问题的人  （比如，为什么我要受苦？） | ❍ | ❍ | ❍ |
| (9) Help with financial support.  慈怀疗护会提供金钱上的帮助。 | ❍ | ❍ | ❍ |
| (10) Applicable only when no further active treatment for underlying medical condition is available. 当找不到更长远的积极医疗方法时，才用慈怀疗护。 | ❍ | ❍ | ❍ |
| (11) Others, please specify: _________________ 其他，请说明：_____________________________ | | | |

**# Palliative Care Information Seeking**

How likely are you to seek information about palliative care from the following media sources?

您有 多大可能 从下面的 媒体消息源 寻找 慈怀疗护的信息？

|  | Extremely Unlikely  完全不可能 | Unlikely  不可能 | Neutral  中立 | Likely  可能 | Extremely likely  完全可能 |
| --- | --- | --- | --- | --- | --- |
| (1) Radio广播 | ❍ | ❍ | ❍ | ❍ | ❍ |
| (2) Television电视 | ❍ | ❍ | ❍ | ❍ | ❍ |
| (3) Newspapers 报纸 | ❍ | ❍ | ❍ | ❍ | ❍ |
| (4) Magazines杂志 | ❍ | ❍ | ❍ | ❍ | ❍ |
| (5) Posters / Pamphlets海报 / 传单 | ❍ | ❍ | ❍ | ❍ | ❍ |
| (6) Search engines on the Internet网上的搜索功能 | ❍ | ❍ | ❍ | ❍ | ❍ |
| (7) Social media (e.g., Facebook, Twitter)  社交媒体（比如脸书、推特） | ❍ | ❍ | ❍ | ❍ | ❍ |

**#Palliative Care Receptiveness**

What is the likelihood that you would consider palliative care?

您会为……考虑慈怀疗护的吗？

|  | Highly unlikely  非常不可能 | Somewhat unlikely  有点不可能 | Neutral  中立 | Somewhat likely  有点可能 | Highly likely  非常可能 |
| --- | --- | --- | --- | --- | --- |
| (1) For yourself  您会为自己考虑慈怀疗护吗？ | ❍ | ❍ | ❍ | ❍ | ❍ |
| (2) For family members  您会为家人考虑慈怀疗护吗？ | ❍ | ❍ | ❍ | ❍ | ❍ |
| (3) For close friends  您会为好朋友考虑慈怀疗护吗？ | ❍ | ❍ | ❍ | ❍ | ❍ |

**# Attitude toward End-of-Life Care Planning**

To what extent do you agree/disagree with the following statements:

下面的几句话，您同意吗？

|  | Strongly disagree  强烈反对 | Disagree  反对 | Neutral  中立 | Agree  同意 | Strongly agree  强烈同意 |
| --- | --- | --- | --- | --- | --- |
| (1) It is most important for me to make end-of life decisions based on my wishes.  在我看来，最重要的是可以按照我自己的想法做临终决定。 | ❍ | ❍ | ❍ | ❍ | ❍ |
| (2) It is important to involve a friend or family member in end-of-life care decisions before a final decision is made.在做最后的临终决定之前，跟朋友或家人商量是重要的。 | ❍ | ❍ | ❍ | ❍ | ❍ |
| (3) If there are conflicts about the end-of-life decisions, my wishes should be given priority. 如果在做临终决定时出现冲突，应该优先考虑我的想法。 | ❍ | ❍ | ❍ | ❍ | ❍ |
| (4) I prefer to have someone close to me make important decisions for me.  我倾向于让我亲近的人帮我做重要的决定。 | ❍ | ❍ | ❍ | ❍ | ❍ |
| (5) It is important to plan for the future.  为将来打算是一件重要的事情。 | ❍ | ❍ | ❍ | ❍ | ❍ |
| (6) Completing an advance care plan will help to ensure that my wishes are followed if I could no longer speak for myself.  如果我不能讲出自己的想法，提早写好遗嘱能够保证我的想法被执行。 | ❍ | ❍ | ❍ | ❍ | ❍ |
| (7) I am willing to complete end-of-life care planning for myself.  我愿意为自己完成临终护理计划。 | ❍ | ❍ | ❍ | ❍ | ❍ |

**# Demographic Profile**

How would you rate your health at the present time?

您如何评价自己目前的健康状况？

❍ Good 好

❍ Fairly good 比较好

❍ Average 一般

❍ Rather poor比较差

❍ Poor差

❍ Cannot judge 无法判断

Which language are you most proficient in? 您最擅长讲什么语言？

❍ English 英文

❍ Chinese华文

❍ Malay马来文

❍ Hindi印度文

❍ Others, please specify: ____ 其他：请说明：__________________

Age年龄

❍ 21-30 21-30岁

❍ 31-40 31-40岁

❍ 41-50 41-50岁

❍ 51-60 51-60岁

❍ 61-70 61-70岁

❍ 71 and above 71 岁及以上

Gender性别

❍ Male 男

❍ Female 女

Ethnicity 种族

❍ Chinese 华族

❍ Malay 马来族

❍ Indian 印度族

❍ Others (please specify:_________) 其他（请说明：_________________）

Your marital status您的婚姻状况

❍ Single 单身

❍ Married 已婚

❍ Separated 分居

❍ Divorced 离婚

❍ Widowed 丧偶

Your highest education level您的最高学历

❍ No formal schooling 无正式教育

❍ PSLE or equivalent 小学四到六年级或等同

❍ GCE O / N Level 新加坡剑桥O水准／N水准

❍ GCE A Level / Diploma 新加坡剑桥A水准／专科文凭

❍ Degree / Higher education 大学文凭／高等教育文凭

Your monthly total household income您的家庭总月收入

❍ Below $1,000 低于$1,000

❍ $1,000-4,999 在$1,000-4,999之间

❍ $5,000-9,999 在$5,000-9,999之间

❍ $10,000-14,999 在$10,000-14,999 之间

❍ $15,000-19,999 在$15,000-19,999 之间

❍ $20,000 and over $20,000或以上

❍ Refuse to answer 拒绝回答

Your housing type您的住房类型

❍ HDB 1– or 2-rooms 一房或两房式政府组屋

❍ HDB 3-rooms 三房式政府组屋

❍ HDB 4-rooms 四房式政府组屋

❍ HDB 5-rooms 五房式政府组屋

❍ HDB Executive / Maisonette 公寓或复式政府组屋

❍ Private apartment/ Condominium/ Landed property私人公寓/公寓楼/房地产
